# A Novel Risk Prediction Model for Severe Acute Kidney Injury in Intensive Care Unit Patients Receiving Fluid Resuscitation

**DOI:** 10.3389/fcvm.2022.840611

**Published:** 2022-04-18

**Authors:** Yunlin Feng, Qiang Li, Simon Finfer, John Myburgh, Rinaldo Bellomo, Vlado Perkovic, Meg Jardine, Amanda Y. Wang, Martin Gallagher

**Affiliations:** ^1^Renal Division, Sichuan Academy of Medical Sciences and Sichuan Provincial People's Hospital, Chengdu, China; ^2^The George Institute for Global Health, University of New South Wales (UNSW), Sydney, NSW, Australia; ^3^Department of Critical Care, University of Melbourne, Melbourne, VIC, Australia; ^4^NHMRC Clinical Trials Centre, University of Sydney, Sydney, NSW, Australia; ^5^Concord Clinical School, University of Sydney, Sydney, NSW, Australia; ^6^South Western Sydney Clinical School, University of New South Wales (UNSW), Sydney, NSW, Australia

**Keywords:** acute kidney injury, risk prediction, model, ICU, fluids resuscitation

## Abstract

**Background:**

To develop a risk prediction model for the occurrence of severe acute kidney injury (AKI) in intensive care unit (ICU) patients receiving fluid resuscitation.

**Methods:**

We conducted a secondary analysis of the Crystalloid vs. Hydroxyethyl Starch Trial (CHEST) trial, a blinded randomized controlled trial that enrolled ICU patients who received intravenous fluid resuscitation. The primary outcome was the first event in a composite outcome of doubling of serum creatinine and/or treatment with renal replacement treatment (RRT) within 28 days of randomization. The final model developed using multivariable logistic regression with backwards elimination was validated internally and then translated into a predictive equation.

**Results:**

Six thousand seven hundred twenty-seven ICU participants were studied, among whom 745 developed the study outcome. The final model having six variables, including admission diagnosis of sepsis, illness severity score, mechanical ventilation, tachycardia, baseline estimated glomerular filtration rate and emergency admission. The model had good discrimination (c-statistic = 0.72, 95% confidence interval 0.697–0.736) and calibration (Hosmer-Lemeshow test, χ^2^ = 14.4, *p* = 0.07) for the composite outcome, with a c-statistic after internal bootstrapping validation of 0.72, which revealed a low degree of over-fitting. The positive predictive value and negative predictive value were 58.8 and 89.1%, respectively. The decision curve analysis indicates a net benefit in prediction of severe AKI using the model across a range of threshold probabilities between 5 and 35%.

**Conclusions:**

Our model, using readily available clinical variables to identify ICU patients at high risk of severe AKI achieved good predictive performance in a clinically relevant population.

## Background

Acute kidney injury (AKI) is common in the intensive care unit (ICU), with a reported incidence of 7–25% for adults (1, 2). The close association between AKI and a range of adverse outcomes, including death, is well accepted, but there are few validated tools to identify ICU patients most at risk of these outcomes (3–5).

The development of consensus definitions for AKI has allowed more consistent diagnosis and comparison between different populations. There are three main AKI definitions in widespread use (6–8) which are broadly similar, using combinations of reductions in urine output, increases in serum creatinine and/or treatment with renal replacement therapy (RRT), to classify the severity of kidney injury. These definitions categorize AKI into stages of increasing severity that correlate with adverse outcomes such as increased mortality and prolonged length of hospital stay (9).

To facilitate early diagnosis and treatment, several models to predict AKI in ICU patients have been proposed (10). However, these models have examined a mixture of approaches (11–15), with data from varying numbers of ICUs and including both randomized trial cohorts and observational cohorts, some of which include specific biomarkers that are not in routine clinical use yet (12–15). In addition, the studied populations are often selected and this may affect the predictive ability and applicability to broader ICU populations of the resultant models. Patients receiving intravenous fluid resuscitation in ICU are common, readily identifiable, and may represent an enriched population at increased risk of severe AKI.

The Crystalloid vs. Hydroxyethyl Starch Trial (CHEST) was a multicenter, prospective, randomized-controlled, clinical trial (RCT) that compared the efficacy and safety of 6% hydroxyethyl starch (HES) (130/0.4) and 0.9% sodium chloride (normal saline) for intravenous fluid resuscitation in patients treated in ICU (16). A key secondary outcome was the incidence and severity of AKI using the Risk, Injury, Failure, Loss and End-stage kidney injury (RIFLE) criteria (7) and the incidence and duration of associated RRT. Using this trial database, we developed a prediction model to determine the risk of developing severe AKI for ICU patients receiving fluid resuscitation.

## Methods

### Study Design

In brief, CHEST was a multicenter, prospective, randomized-controlled, clinical trial (RCT) that compared the use of HES and saline for intravenous fluid resuscitation in patients in 32 ICUs in Australia and New Zealand (Clinicaltrials.gov identifier#: NCT00935168). Detailed descriptions of the study protocol, statistical analysis plan and results have been published previously (16, 17). The CHEST study was approved by the human research Ethics Committee of Northern Sydney Central Coast Health (AU RED Ref: HREC/09/HARBR/14), and by each participating institution. The present study is a *post-hoc* analysis of the CHEST database with the objective of developing a model for the prediction of severe AKI in ICU patients receiving fluid resuscitation.

### Study Outcomes

The primary outcome of the model is severe AKI, defined as the first event in a composite outcome incorporating doubling of serum creatinine (from the pre-randomization value) and/or treatment with RRT within 28 days of randomization. It is important to note that this outcome differs from the reported AKI outcome in the primary CHEST article, which used the RIFLE criteria (17) defined by changes in either urine output or serum creatinine from randomization.

### Demographic and Clinical Variables

In order to minimize selection bias, our analysis included all patients from the original study population for whom consent to use of their data was obtained and were not lost to follow-up. Demographic and clinical variables were collected as described previously (16), and variables collected immediately before randomization were deemed as baseline variables. We examined 17 candidate baseline variables that were prospectively considered by the authors to be potential indicators of AKI risk: age, sex, weight, heart rate (HR), central venous pressure (CVP), mean arterial pressure (MAP), urine output during 6 h prior to randomization, serum creatinine (serum Cr), estimated glomerular filtration rate (eGFR), serum lactate concentration, Acute Physiology and Chronic Health Evaluation (APACHE) II score (18), Sequential Organ Failure Assessment (SOFA) score for the cardiovascular system (19), the presence of sepsis (20), the presence of trauma, source of admission, treatment with mechanical ventilation, and the nature of ICU admission (surgical or nonsurgical). As the allocated study treatment (HES or saline) was assigned at randomization, this variable was not considered as a baseline variable and excluded from multivariate regression models.

eGFR was calculated using the Chronic Kidney Disease Epidemiology Collaboration (CKD-EPI) equation (21). The source of admission was defined as the place from where the patients had been transferred to ICU and was classified as: hospital floor, emergency department, operating room following elective surgery, operating room following emergency surgery, and other hospitals. Mechanical ventilation included either invasive ventilation via an endotracheal tube or non-invasive respiratory support via a mask or other non-invasive interface.

### Statistical Analysis

As the analysis focused on the baseline clinical variables, all patients in the baseline analysis of the original study publication with complete demographic data and information about the study outcome were included. Continuous variables were presented as median (interquartile range) or mean ± standard deviation (SD), categorical variables were presented as number (percentage). APACHE II score was considered as a continuous variable. Univariable regression was used to examine the relationship between candidate predictors and the study outcome. Multivariable logistic regression model was used to identify independent risk factors for the study outcome.

For modeling, we followed a three-step procedure. First, we developed a primary model by multivariable logistic regression using a backward elimination approach (threshold *p* < 0.05) to select variables (22) after excluding variables that would potentially have high risk of in the development population. Possible first-order interactions were also explored unavailability in real-world clinical practice based on their proportions of missing data and kept in the model if statistically significant (*p* < 0.05). Second, we developed a secondary model by removing interaction terms in the primary model and compared the performance of these two models using c-statistics (equal to the area under the receiver-operating characteristic curve). If no difference between these primary and secondary models was found, the secondary model was preferred due to its greater simplicity and avoidance of over-fitting, consistent with the Transparent Reporting of a multivariable prediction model for Individual Prognosis Or Diagnosis (TRIPOD) statements (23).Third, we simplified the model from step two by removing variables that were not statistically significant and tested the performance of the final parsimonious model. The three models were compared, and the final model was chosen as the best balance between calibration, discrimination and clinical practicability. To test the performance of the model, discrimination was assessed by c-statistics (24), and calibration was assessed by Hosmer-Lemeshow test (25) and calibration plot.

In addition we performed two sensitivity analyses. The first tested whether forcing the randomization treatment (HES or normal saline) into the multivariable regression model, altered the model's predictive performance, and the second examined whether the timing of the primary outcome, at 7 days rather than 28 days from randomization altered model performance to be consistent with current diagnostic criteria of AKI.

For validation, we randomly sampled from the study dataset using the bootstrapping method (26) (*n* = 10,000 replications) to evaluate the over-optimism inherent in the final model. Confidence intervals of the over-fitting in c-statistics for the bootstrap corrections were computed based on the assumption of normal distributions. The bootstrap-adjusted performance was calculated and the final risk prediction model was translated into a predictive equation. Positive predictive value (PPV) and negative predictive value (NPV) were calculated, and a decision curve analysis was performed to evaluate the net benefit of using the model in predicting the primary outcome across a range of threshold probabilities.

The reporting of this prognostic model study followed the TRIPOD statement (23). The risk of bias of the final model was assessed using the Prediction Model Risk of Bias Assessment Tool (PROBAST) (27). In addition, we used the final model and REDCap electronic data capture tools (28) securely hosted at Sichuan Provincial People's Hospital to develop a web-based risk assessment tool accessible to readers.

All analyses were performed using SAS/STAT software v.9.1 (SAS Institute Inc., Cary, NC, USA) with statistical significance set at *P* < 0.05.

## Results

### Baseline Characteristics and Study Outcome

Baseline variables and the study outcome in the study population are listed in Table 1. Of the 6,742 patients reported in the original publication, 15 patients were excluded because of missing RRT follow up data, leaving 6,727 patients for analysis. The majority of the 6,727 patients entered ICU from the operating room or emergency department. Data for urine output during 6 h prior to randomization, baseline CVP and baseline serum lactate were available for only 2,802, 2,300 and 5,555 patients respectively, so these three variables were excluded from modeling (see Table 1). The proportion of individuals with at least one variable with missing value was 2.79% (188/6727).

**Table 1:** Baseline characteristics and the study outcome of the study population.

**Variables**		Summary values^*^	Number of values in dataset ^**^
**Demographic**			
Age (years)		63.1 ± 16.9 (6,727)	6,727
Male		4,060 (60.4%)	6,726
**Renal parameters**			
serum Cr (μmol/L)		100.5 ± 57.2	6,639
eGFR (ml/min/1.73 m^2^)		73.8 ± 30.8	6,637
Urine output 6 h before randomization (ml)		440.2 ± 420.7	2,802
**Other clinical parameters**			
HR (beats per minute)		89.0 ± 23.4	6,691
Weight (kg)		78.9 ± 20.9	6,727
CVP (mmH_2_O)		9.2 ± 5.3	2,300
MAP (mmHg)		73.8 ± 14.8	6,687
Lactate (mmol/L)		2.0 ± 1.76	5,555
APACHE II score		17.9 ± 7.6	6,688
Cardiovascular SOFA score	0	1,172 (17.5%)	6,698
	1	2,415 (36.1%)	6,698
	2	40 (0.6%)	6,698
	3	2,161 (32.3%)	6,698
	4	910 (13.6%)	6698
Presence of sepsis		1,936 (28.8%)	6,724
Presence of Trauma		528 (7.9%)	6,727
Presence of nonsurgical diseases		3,844 (57.2%)	6,716
Admission Source to ICU	Hospital floor	1,323 (19.7%)	6,723
	Emergency department	1,857 (27.6%)	6,723
	OR following elective surgery	1,574 (23.4%)	6,723
	OR following emergency surgery	1,254 (18.7%)	6,723
	Other hospitals (ICU or non-ICU%)	715 (10.6%)	6,723
Mechanical ventilation		4,307 (64.5%)	6,679

*APACHE II, Acute Physiology and Chronic Health Evaluation II; CVP, central venous pressure; eGFR, estimated glomerular filtration rate; HES, hydroxyethyl starch; MAP, mean arterial pressure; OR, operation room; RRT, renal replacement therapy, serum Cr, serum creatinine; SD, standard deviation; SOFA, Sequential Organ Failure Assessment*.

* *Values were expressed as mean ± standard deviation for continuous variables or number (percentage) for categorical variables*.

** *Some numbers were less than the total number (n = 6,727) of the study population due to missing data*.

**Figure 1:**
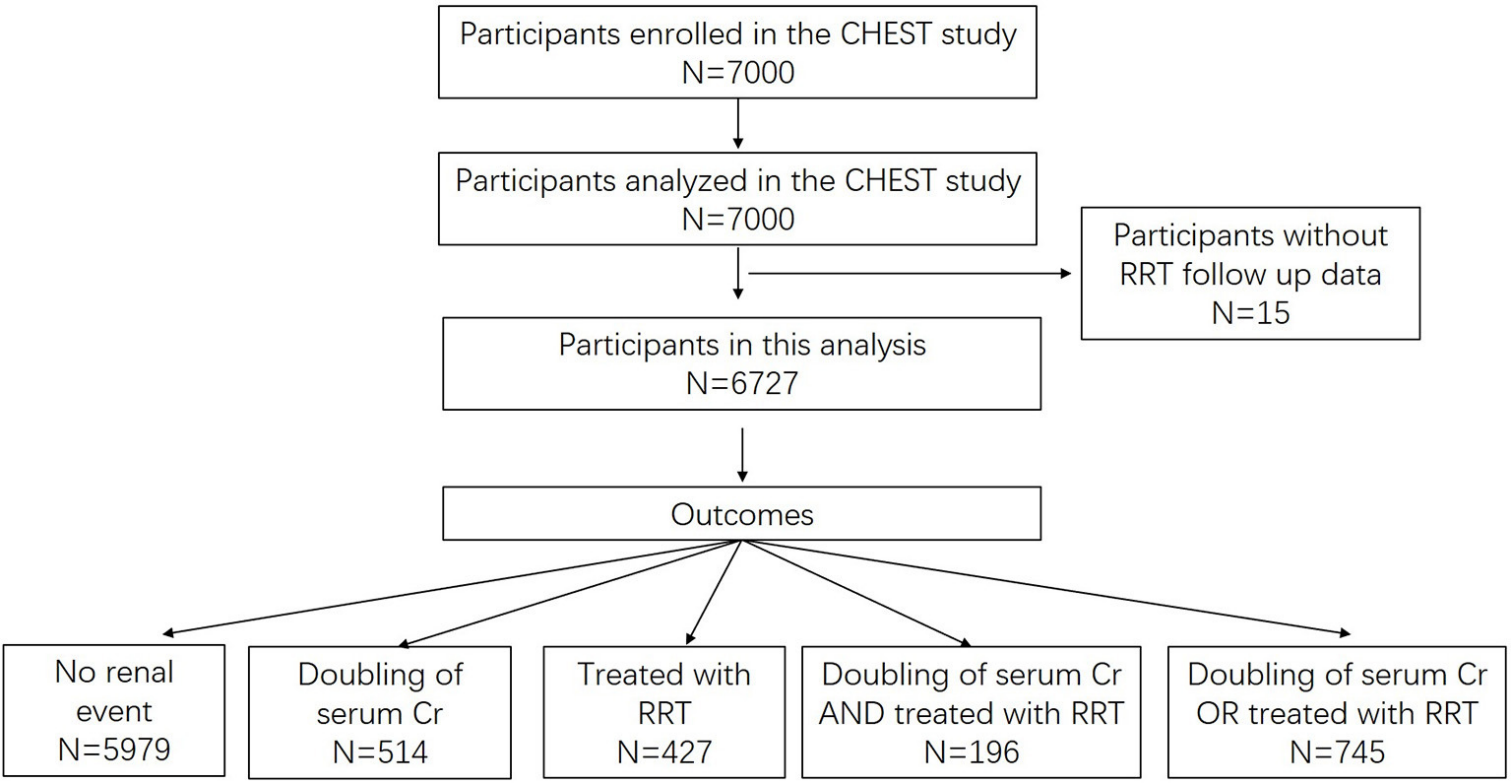
Participant flow diagram. Cr, creatinine; RRT, renal replacement treatment.

Within 28 days after randomization, 514 (7.6%) participants experienced a doubling of serum Cr, 427 (6.4%) were treated with RRT and 196 (2.9%) experienced both events. In total 745 (11.1%) participants developed the study outcome (Figure 1).

### Univariate Analysis of Candidate Predictors

The univariate relationships of all candidate model variables with the study outcome are presented in Table 2. The majority of these variables were associated with the study outcome, but sex, MAP, admission following emergency surgery (compared to admission from emergency department) and mechanical ventilation were not.

**Table 2:** Univariate odds ratios of candidate predictive variables for the study outcome.

**Variables**		**Odds ratios**	**95% CI**	***P*-value**
Male^†^		0.95	0.82–1.11	0.5375
Age per 5 years increase		1.03	1.01–1.06	0.0084^*^
Baseline eGFR per 5 ml/min/1.73 m^2^ decrease		1.08	1.06–1.09	0.0000^*^
HR per 5 bpm increase		1.11	1.09–1.13	0.0000^*^
Weight per 5 kg increase		1.02	1.01–1.04	0.0080^*^
MAP per 10 mmHg increase		1.02	0.97–1.08	0.4367
APACHE II score		1.06	1.05–1.07	0.0000^*^
Cardiovascular SOFA score		0.83	0.71–0.97	0.0172^*^
Presence of sepsis		2.42	2.07–2.83	0.0000^*^
Presence of Trauma		0.53	0.37–0.76	0.0005^*^
Presence of nonsurgical diseases		2.09	1.31–3.33	0.0019^*^
Admission source^#^	Hospital floor	1.55	1.25–1.91	0.0000^*^
	OR after elective surgery	0.55	0.42–0.70	0.0000^*^
	OR after emergency surgery	1.04	0.83–1.31	0.7086
	Other hospital	1.46	1.13–1.88	0.0038^*^
Mechanical ventilation at admission		1.16	0.99–1.37	0.0727

*Serum Cr was not included in the analysis due to its high correlation with eGFR. Continuous APACHE II score and Cardiovascular SOFA score were used*.

*APACHE II, Acute Physiology and Chronic Health Evaluation II; bpm, beats per minutes; CVP, central venous pressure; eGFR, estimated glomerular filtration rate; HES, hydroxyethyl starch; HR heart rate, -MAP, mean arterial pressure; OR, operating room; RRT, renal replacement therapy, serum Cr, serum creatinine; SOFA, Sequential Organ Failure Assessment*.

† *vs. female*.

# *vs. admission from the emergency department*.

* *Statistical significance*.

### Prediction Model

**Figure 2:**
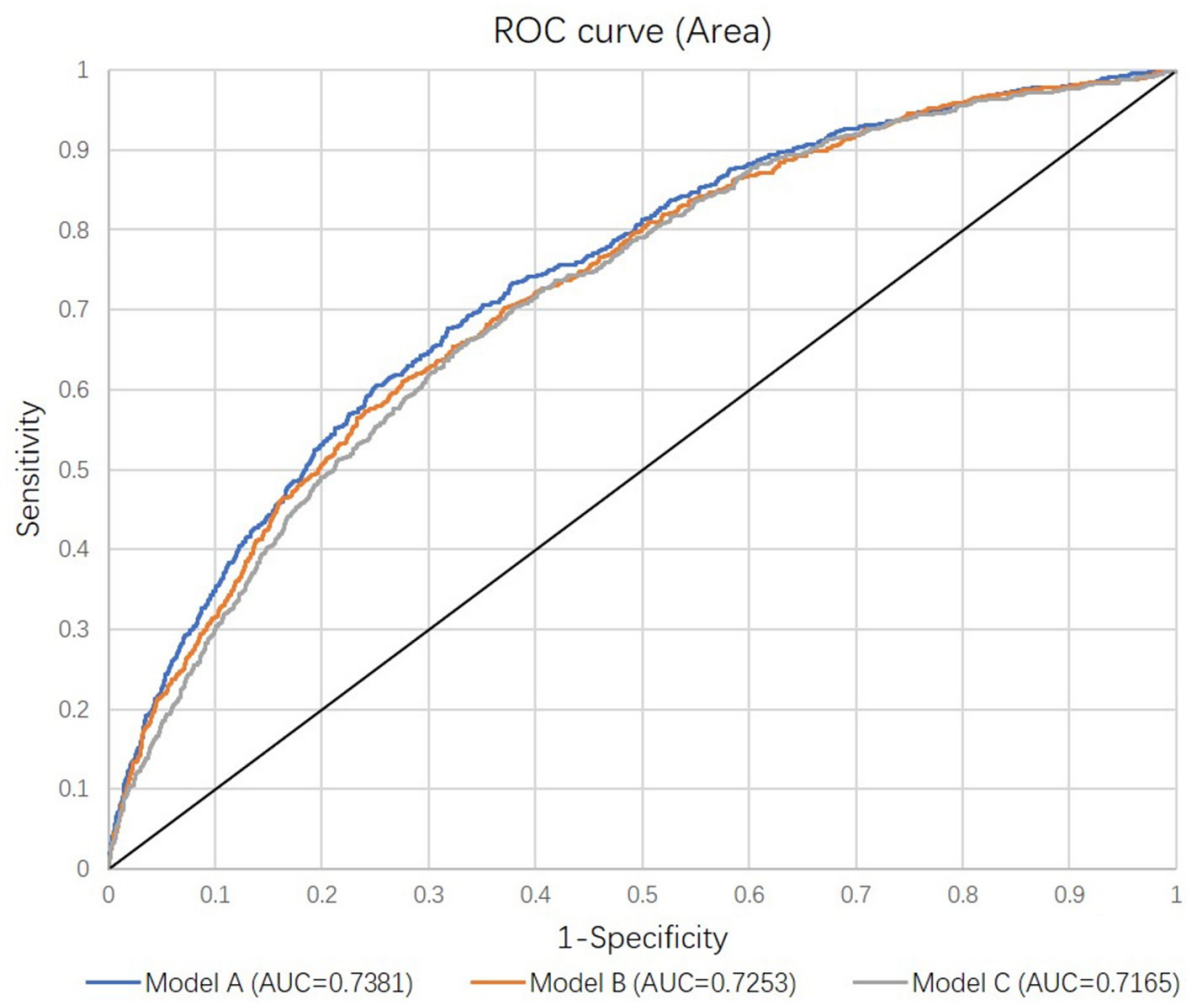
Receiver Operating Curves comparing the discrimination of the three models. Each model was adjusted for randomly assigned treatments. See details in Methods.

Evaluated variables in each model were examined and compared (Supplementary Table S1). In addition to stand-alone significant variables including age, baseline eGFR, heart rate, APACHE II score, sepsis, mechanical ventilation at admission, and admission source, the primary model (Model A) also included eight interactive terms as significant factors. However, the performance of the secondary model (Model B) after removing interactive terms did not materially differ from the primary model (Figure 2). After removing these interactive terms, seven variables became non-significant, resulting in the final model (Model C) including six significant predictors (Table 3). The comparison of all three models indicated no meaningful difference in C-statistics for the primary, secondary and final models (Figure 2).

**Table 3:** Odds ratios of independently significant predictors in the final model.

**Variables**		**Doubling of serum Cr or RRT within 28 days after randomization**		
		**Odds ratios**	**95% CI**	***P*-value**
Baseline eGFR per 5 ml/min/1.73 m^2^ decrease		1.052	1.037–1.067	<0.0001
HR per 5 bpm increase		1.084	1.065–1.103	<0.0001
APACHE II score		1.039	1.027–1.052	<0.0001
Presence of sepsis		1.580	1.325–1.885	<0.0001
MV at admission		1.242	1.032–1.491	0.02
Admission source^*^	Hospital floor	1.455	1.166–1.814	0.009
	OR after elective surgery	1.231	0.922–1.644	
	OR after emergency surgery	1.294	1.009–1.659	
	Other hospitals	1.448	1.103–1.900	

*Continuous APACHE II score was used*.

*APACHE II, Acute Physiology and Chronic Health Evaluation II; bpm, beats per minutes; eGFR, estimated glomerular filtration rate; HR, heart rate; MV, mechanical ventilation; OR, operating room; RRT, renal replacement therapy*.

* *vs. admission from the emergency department*.

The sensitivity analysis of including randomization treatment in the final model showed no change in the effect of the six predictors or the C-statistic (Table 4). The sensitivity analysis of the ability of the final model to predict doubling of serum creatinine and RRT within 7 days after randomization again showed no change in the effect of the model variables indicated all predictors remained significant except for mechanical ventilation at admission (Supplementary Table S2).

**Table 4:** Sensitivity analysis of including randomization treatment in the multivariable regression model.

**Variables**		**Doubling of serum Cr or RRT within 28 days after randomization**					
		**Final model**			**Model with treatment forced in**		
		**Odds ratios**	**95% CI**	***P*-value**	**Odds ratios**	**95% CI**	***P*-value**
Randomization treatment (HES) ^*^				1.177	1.002–1.382	0.047
Baseline eGFR per 5 ml/min/1.73 m^2^ decrease	1.052	1.037–1.067	<0.0001	1.052	1.037–1.067	<0.0001
HR per 5 bpm increase	1.084	1.065–1.103	<0.0001	1.084	1.065–1.103	<0.0001
APACHE II score	1.039	1.027–1.052	<0.0001	1.039	1.027–1.052	<0.0001
Presence of sepsis	1.580	1.325–1.885	<0.0001	1.580	1.325–1.885	<0.0001
MV at admission	1.242	1.032–1.491	0.020	1.242	1.033–1.493	0.021
Admission source^†^	Hospital floor	1.455	1.166–1.814	0.009	1.456	1.167–1.815	0.094
	OR after elective surgery	1.231	0.922–1.644		1.233	0.923–1.647	
	OR after emergency surgery	1.294	1.009–1.659		1.292	1.008–1.656	
	Other hospitals	1.448	1.103–1.900		1.442	1.099–1.892	
	AUC (95% CI)		0.717 (0.697, 0.736)			0.715 (0.696, 0.735)

* *vs. saline*.

† *vs. admission from the emergency department*.

*APACHE II, Acute Physiology and Chronic Health Evaluation II; bpm, beats per minutes; CI, confidence interval; eGFR, estimated glomerular filtration rate; HR, heart rate; MV, mechanical ventilation; OR, operating room; RRT, renal replacement therapy*.

### Final Model Performance and Internal Validation

**Figure 3:**
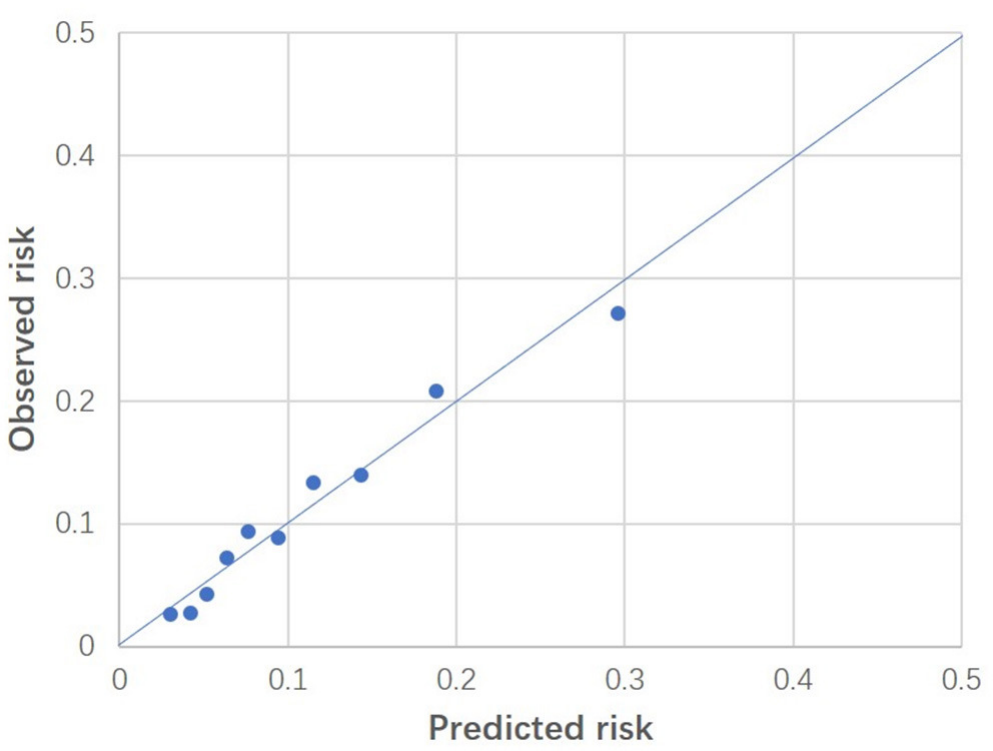
Calibration plot of the final model. Predicted risk was indicated on horizontal axis, whereas observed risk was indicated on vertical axis. These results were based on patients grouped into deciles of predicted risk. Modified Hosmer-Lemeshow test indicated good fitness (*p* = 0.07).

**Figure 4:**
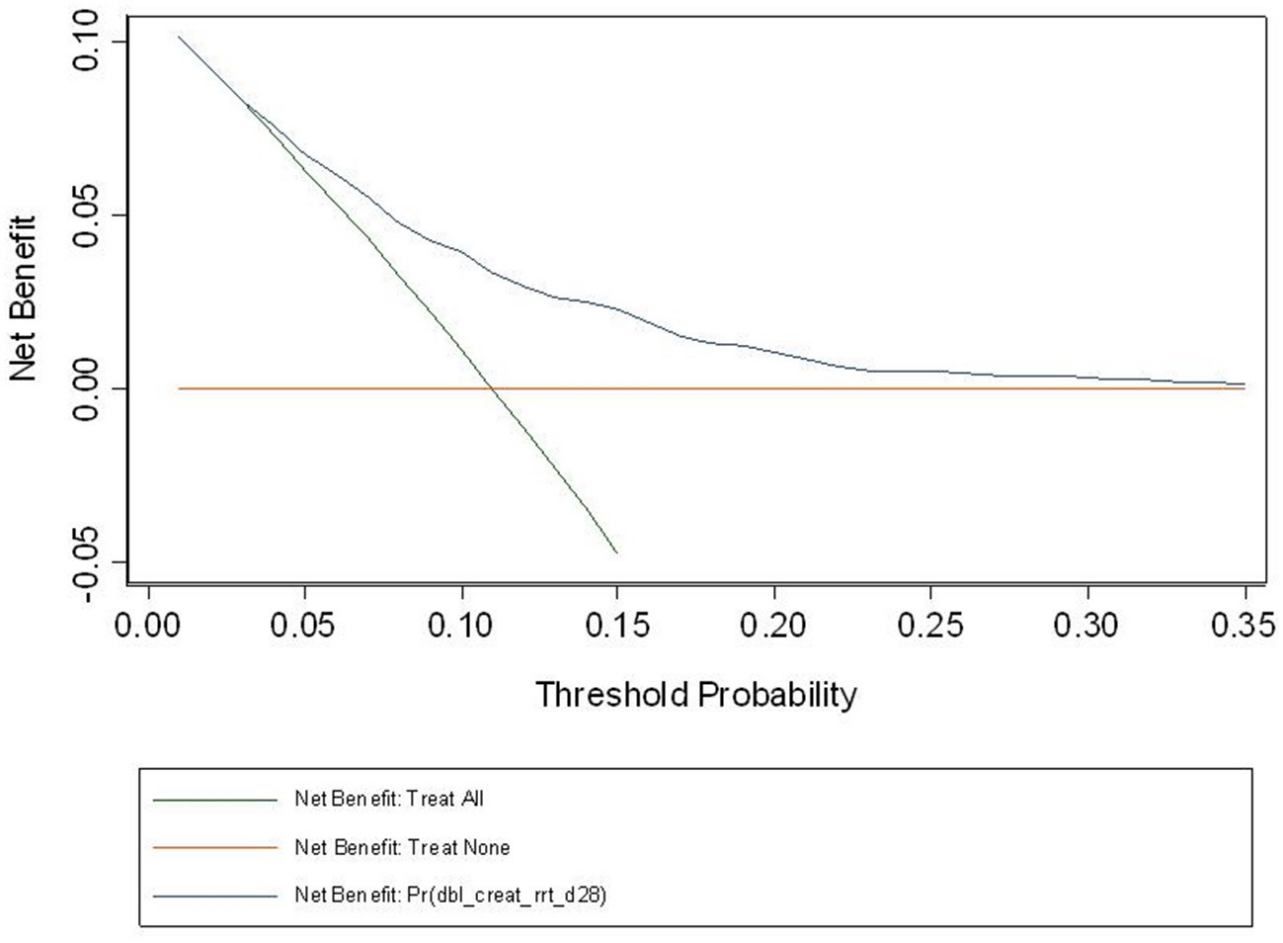
Decision curve based on the final model. Decision curve analysis is a relatively recent approach, seeking to overcome the limitation of the usual model assessment tools such as calibration and discrimination in their clinical application. If one doesn't use the model, then any intervention could be applied to everyone (intervention for all) or no-one (intervention for none). Between these two interventions sits the impact of the model, and the fact that our curve sits above the intersection of both curves, across the range of probabilities for AKI in our study population, suggest that the model will be of net benefit in this population. At a population level, the net benefit of the intervention treatment will be realized across a larger number of patients across the spectrum of risk.

The observed and predicted risk of the composite primary outcome based on the final model were similar, with a test of goodness of fit indicating good calibration (modified Hosmer-Lemeshow test, χ^2^ = 14.4, *p* = 0.07) (Figure 3). Internal validation using the bootstrap method revealed the degree of over-optimism on c-statistics of the final prediction model was 0.0055 (95% CI, −0.0129 to 0.0240), resulting in an equivalent c-statistic after bootstrap validation of 0.711. The PPV and NPV of the final model to predict severe AKI in ICU patients receiving fluid resuscitation within 28 days from admission were 58.8 and 89.1%, respectively. The decision curve analysis indicates a net benefit in prediction of severe AKI using the model across a range of threshold probabilities between 5 and 35% (Figure 4), illustrating the additional benefit of the model in predicting severe AKI over and above the approaches of “intervention for all” and “intervention for none”.

### Risk of Bias Assessment of the Final Model

According to the PROBAST assessment, 19 of 20 signaling questions were rated as “Yes,” and 1 was rated as “Probably Yes,” thus the overall risk of bias of the final model was rated as low risk of bias. Detailed rationales of answers were described in Supplementary Table S3.

### Risk Predictive Equation for the Study Outcome

From the above computation, the following risk prediction equation was derived:


(1) $$\begin{array}{r} {\text{P}}_{\text{outcome}}=1/\left(1+\exp\left(-A\right)\right) \end{array}$$

where P_outcome_ indicates the probability for the study outcome occurring, and A = −0.0101 ^*^ eGFR + 0.0161 ^*^ Heart Rate + 0.0378^*^APACHE II score + 0.4577^*^ (if sepsis) + 0.3747^*^ (if admitted from hospital floor) + 0.2080^*^ (if admitted from OR after elective surgery) + 0.2577^*^ (if admitted from OR after emergency surgery) + 0.3699^*^ (if admitted from other hospitals) + 0.2154^*^ (if mechanical ventilation on admission) – 4.1529.

The prediction model was translated using REDCap to an online calculator for readers' convenience (to visit, please scan the QR code in Supplementary Figure S1 using mobile devices or visit: http://redcap.scrds.net/surveys/ and enter code: 7KMH8HN7N). The probability would be shown automatically at the completion of all predictors. Readers are encouraged to submit their data that can be collected for further improvement of the model.

## Discussion

We report the development of a model for predicting severe AKI risk in a population of ICU patients who received fluid resuscitation, using data from a large, multi-center randomized trial. The final model includes six significant clinical predictors: admission diagnosis of sepsis, illness severity score, mechanical ventilation, tachycardia, baseline estimated glomerular filtration rate and emergency admission; all of which are readily evaluable close to the point of ICU admission and, together, demonstrate robust predictive ability, discrimination and calibration.

Our findings broadly accord with those of other similar recent studies (12, 15, 29, 30), but also highlight some important challenges in this field. Flechet et al.'s model (12) is the closest in design to our analysis, being a randomized trial dataset collected across 7 centers (the Early Parenteral Nutrition Completing Enteral Nutrition in Adult Critically Ill Patients Study, EPaNIC), and both models share a number of predictive variables such as baseline renal function, requirement for emergency surgery and clinical suspicion of sepsis at baseline. Flechet's report included several models defined by different time points in patients' hospital stay, with their “admission model” the most analogous to our final model. Its discriminative ability for AKI within the first week of ICU stay was similar to our finding (c statistic of 0.75), and improved when additional variables from the first 24 h following admission to ICU were included (c statistic rising to 0.82). The underlying populations had some important differences, with the EPaNIC study having a higher proportion of patients entering ICU following elective cardiac surgery and a 30% lower mortality rate, but similar rates of renal replacement therapy use, compared to the CHEST population (16, 31).

Malhotra and colleagues used prospective data from a single center to derive a model and validated it using data from another US facility (29). This model included several chronic disease conditions along with acute risk factors such as acidosis, treatment with mechanic ventilation and the presence of sepsis. The discrimination of this model was high in both development and validation cohorts, but the inclusion of data from the first 48 h of participants ICU stay likely played a part in this, as Flechet et al. also saw increases in the c statistic as data from later in the patient journey was added to their admission model (12). Koyner et al.'s model, which included both general hospital and ICU patients, using extensive electronic health record data, had a better discrimination compared with our model, which may be in part due to greater number of variables and the use of data from after the patients' admission (30).

Our model benefits from the prospective nature of the CHEST study, which examined a large, clearly defined cohort of general ICU patients, was successful in recruiting patients within a median of 12 h of ICU admission, and uses data from a large number of centers. The resultant final model is relatively simple, enhancing usability and reducing the risk of model over-fitting, and estimates a probability for more severe AKI in the 28 days after baseline. For example, a septic patient with a baseline eGFR of 30 ml/min/1.73 m^2^, heart rate of 90, APACHE II score of 18 who has been transferred to ICU from the hospital floor on mechanical ventilation, the risk of developing the composite event (doubling of serum Cr or treatment with RRT) in the following 28 days would be 21.8%. However, external validation of these findings is an important next step.

Our modeling is the first in this area to use decision curve analysis, first described in 2006 (32), as a tool to understand the “net benefit” of a predictive model compared to strategies that clinically act upon all or none of defined patient groups. A challenge in interpreting these results is understanding the nature of clinical responses to a severe AKI diagnosis, where there is an absence of effective treatments proven to reduce the occurrence, or outcomes, of severe AKI. However, identification of populations at high risk of severe AKI may assist in the prognostic enrichment of clinical trials that test treatments or strategies to prevent AKI, and could be used to assess baseline balance in risk of developing AKI in clinical trials where AKI is a trial endpoint. Additionally, our model may also have value in smaller intensive care units, where RRT support is limited, by aiding timely decisions to transfer patients at higher risk to centers with greater capacity to treat AKI. As such, the low risk of harms from applying the model and responding to a diagnosis of severe AKI, would suggest the threshold probability for its use is at the lower end of the presented range, where the net benefit is most pronounced.

Our study has limitations. First, the study population was derived from an existing clinical trial dataset and, whilst the inclusion criteria for the CHEST study were broad, it excluded important patient groups such as those with advanced AKI at baseline and children. Second, a relatively small proportion of the patients developed the study outcome, accounting for 11.1% of the original population, which was lower than the average reported prevalence of AKI in general ICU population (1, 2), and was likely a function of study entry criteria. Third, the prediction model did not include urine output, which is a part of most definitions for AKI, as the data were incomplete for a large proportion of the study population; an issue also seen in other published models (10, 29). Fourth, the PPV is relatively low, indicating that we are less confident in predicting a patient who would develop AKI in 28 days than in ruling out a patient who would not develop the same outcomes. Fifth, it needs to be borne in mind that the dataset underlying this model includes 620 deaths that did not experience the study outcome, which mostly occurred within the first 10 days of the study. Finally, external validation is central to fully understanding the value of our model.

## Conclusions

We have developed a novel risk prediction model for severe acute kidney injury in a population of general ICU patients receiving fluid resuscitation. The final model includes six significant predictors and has good discrimination and calibration for the composite outcome of doubling of serum creatinine or treatment with RRT within 28 days. External validation is needed to explore its generalizability.

## Data Availability Statement

The original contributions presented in the study are included in the article/Supplementary Material, further inquiries can be directed to the corresponding author.

## Ethics Statement

The studies involving human participants were reviewed and approved by human research Ethics Committee of Northern Sydney Central Coast Health. The patients/participants provided their written informed consent to participate in this study.

## Author Contributions

JM, SF, MJ, VP, AW, and MG conceived the study. QL, YF, and MJ did the data analyses. The article was written by YF, with input from all co-authors. All authors interpreted the results, contributed to the critical revising of the manuscript, and have approved the final version.

## Funding

The CHEST study was funded by a project grant from the National Health and Medical Research Council of Australia and by unrestricted grants from the New South Wales Ministry of Health and Fresenius Kabi, the manufacturer of Voluven^®^. FYL is supported in part by an Australian Government Research Training Program Scholarship (RTP) for study toward a Ph.D. in the Faculty of Medicine of UNSW, Australia. AW is supported by RACP Jacquot Research Establishment Fellowship, Australia. Funding agencies had no input into the design, conduct, data collection, statistical analysis, or writing of the manuscript.

## Conflict of Interest

The authors declare that the research was conducted in the absence of any commercial or financial relationships that could be construed as a potential conflict of interest.

## Publisher's Note

All claims expressed in this article are solely those of the authors and do not necessarily represent those of their affiliated organizations, or those of the publisher, the editors and the reviewers. Any product that may be evaluated in this article, or claim that may be made by its manufacturer, is not guaranteed or endorsed by the publisher.
