# Supplementary material for: A Novel Risk Prediction Model for Severe Acute Kidney Injury in Intensive Care Unit Patients Receiving Fluid Resuscitation
Source: Front Cardiovasc Med. 2022 Apr 18;9:840611. doi: 10.3389/fcvm.2022.840611 (PMC9058114; doi:10.3389/fcvm.2022.840611)
Supplement: Supplementary file 1 [file Data_Sheet_1.docx]

**Supplementary**

Supplemental Digital Content – Table S1. Evaluated variables in different models based on multivariable logistic regression assessment.

Supplemental Digital Content – Table S2. Sensitivity analysis for doubling of serum creatinine or RRT within 7 days after randomization

Supplemental Digital Content – Table S3. Risk of bias assessment based on PROBAST tool.

Supplemental Digital Content – Figure S1. QR code for an online calculator based on the prediction model.

Supplementary Table S1. Evaluated variables in different models based on multivariable logistic regression assessment.

| Evaluated variables | Primary model | Secondary Model | Final Model |
| --- | --- | --- | --- |
| Age (years) | √ | √ |  |
| Baseline eGFR§ | √ | √ | √ |
| HR† | √ | √ | √ |
| APACHE II score | √ | √ | √ |
| Presence of sepsis | √ | √ | √ |
| MV at admission | √ | √ | √ |
| Admission source‡ | √ | √ | √ |
| Inclusion criteria of heart rate >90 bpm | √ | √ |  |
| Inclusion criteria of Capillary refill time > 1 s | √ | √ |  |
| Inclusion criteria of urine output < 0.5 ml/kg/h | √ | √ |  |
| Trauma at baseline | √ | √ |  |
| Primary diagnosis of medical or surgical | √ | √ |  |
| SOFA Cardiovascular component | √ | √ |  |
| Age * Trauma | √ |  |  |
| Baseline eGFR * Capillary refusion time > 1s | √ |  |  |
| Baseline eGFR * Urine output < 0.5 ml/kg/h | √ |  |  |
| Baseline eGFR * Presence of nonsurgical diagnosis | √ |  |  |
| APACHE II score * Admission resource | √ |  |  |
| APACHE II score * Presence of nonsurgical diagnosis | √ |  |  |
| Capillary refusion time > 1s * MC at admission | √ |  |  |
| MV at admission*SOFA Cardiology score | √ |  |  |
| AUC (95% CI) | 0.738 (0.719, 0.757) | 0.725 (0.706, 0.745) | 0.717 (0.697, 0.736) |

§ per 5 ml/min/1.73m^2^ decrease; † per 5 beats/minute increase; ‡ potential admission source versus emergency department included hospital floor, operation room after elective surgery, operation room after emergency surgery and other hospitals.

Abbreviations: APACHE II, Acute Physiology and Chronic Health Evaluation II; eGFR, estimated glomerular filtration rate; HR, heart rate; MV, mechanical ventilation; SOFA, Sequential Organ Failure Assessment.

Supplemental Digital Content – Table S2. Sensitivity analysis for doubling of serum creatinine or RRT within 7 days after randomization

|  | | Doubling of serum Cr or RRT within 28 days after randomization | | | Doubling of serum Cr or RRT within 7 days after randomization | | |
| --- | --- | --- | --- | --- | --- | --- | --- |
| Variables | | Odds ratios | 95% CI | P-value | Odds ratios | 95% CI | P-value |
| Baseline eGFR per 5 ml/min/1.73m^2^ decrease | | 1.052 | 1.037-1.067 | <.0001 | 1.054 | 1.039-1.070 | <.0001 |
| HR per 5 bpm increase | | 1.084 | 1.065-1.103 | <.0001 | 1.085 | 1.066-1.105 | <.0001 |
| APACHE II score | | 1.039 | 1.027-1.052 | <.0001 | 1.037 | 1.025-1.050 | <.0001 |
| Presence of sepsis | | 1.580 | 1.325-1.885 | <.0001 | 1.560 | 1.299-1.874 | <.0001 |
| MV at admission | | 1.242 | 1.032-1.491 | 0.020 | 1.163 | 0.961-1.407 | 0.121 |
| Admission source* | Hospital floor | 1.455 | 1.166-1.814 | 0.009 | 1.446 | 1.147-1.822 | 0.006 |
|  | OR after elective surgery | 1.231 | 0.922-1.644 |  | 1.280 | 0.945-1.732 |  |
|  | OR after emergency surgery | 1.294 | 1.009-1.659 |  | 1.414 | 1.093-1.828 |  |
|  | Other hospitals | 1.448 | 1.103-1.900 |  | 1.538 | 1.160-2.039 |  |
| AUC (95% CI) | | 0.717 (0.697, 0.736) | | | 0.713 (0.693, 0.733) | | |

Abbreviations: APACHE II, Acute Physiology and Chronic Health Evaluation II; bpm, beats per minutes; CI, confidence interval; eGFR, estimated glomerular filtration rate; HR, heart rate; MV, mechanical ventilation; OR, operating room; RRT, renal replacement therapy.

* versus admission from the emergency department

Supplemental Digital Content – Table S3. Risk of bias assessment based on PROBAST tool.

| **Question** | **Answer** | **Rationale** |
| --- | --- | --- |
| **Domain 1: Participants** |  |  |
| 1·1 Were appropriate data sources used, e.g., cohort, RCT or nested case-control study data? | Yes | Data from RCT design was used. |
| 1·2 Were all inclusions and exclusions of participants appropriate? | Yes | Patients with complete demographic data and available information about the study outcome were included in the analysis. This was due to data availability. Besides, patients without renal follow-up were excluded since this was a study on renal outcomes. |
| **Overall risk of bias of Domain 1** | **Low risk of bias** |  |
| **Domain 2: Predictors** |  |  |
| 2·1 Were predictors defined and assessed in a similar way for all participants? | Yes | All predictors were objectively measured. Diagnosis of coexisting conditions were based on well-established criteria. |
| 2·2 Were predictor assessments made without knowledge of outcome data? | Yes | All predictors were collected at patients admission. |
| 2·3 Are all predictors available at the time the model is intended to be used? | Yes | All predictors in the final model were easy to acquire, including patient baseline characteristics and laboratory tests. |
| **Overall risk of bias of Domain 2** | **Low risk of bias** |  |
| **Domain 3: Outcome** |  |  |
| 3·1 Was the outcome determined appropriately? | Yes | The primary outcome of the intended model is severe AKI, defined as the first event in a composite outcome incorporating doubling of serum creatinine and treatment with RRT within 28 days of randomization. These are objectively measured results. |
| 3·2 Was a pre-specified or standard outcome definition used? | Yes | The composite outcome was pre-specified. |
| 3·3 Were predictors excluded from the outcome definition? | Yes | The predictors were independent from outcome definition. |
| 3·4 Was the outcome defined and determined in a similar way for all participants? | Yes | The composite outcome was objective. |
| 3·5 Was the outcome determined without knowledge of predictor information? | Yes | The outcome was independent from predictors. |
| 3·6 Was the time interval between predictor assessment and outcome determination appropriate? | Yes | All predictors included in the final model were assessed at ICU admission. The time interval between predictor assessment and outcome determination is sufficient. |
| **Overall risk of bias of Domain 3** | **Low risk of bias** |  |
| **Domain 4: Analysis** |  |  |
| 4·1 Were there a reasonable number of participants with the outcome? | Yes | The total number of candidate variables was 17, and the number of events was 745, so the number of events per variable (EPV) =745/17≈44, which was reasonable. |
| 4·2 Were continuous and categorical predictors handled appropriately? | Yes | No categorization was applied to continuous variables. |
| 4·3 Were all enrolled participants included in the analysis? | Yes | All eligible patients were included in the analysis. |
| 4·4 Were participants with missing data handled appropriately? | Probably yes | The proportion of individuals with at least one variable with missing value was 2.7% (188/6727). We have tried single imputation stratified by median age group and gender. The results still hold and the resulting AUC=0.709 (95%CI 0.690-0.728), so we consider that CCA remains the preferred modeling approach. |
| 4·5 Was selection of predictors based on univariable analysis avoided? | Yes | Univariable analysis was done to investigate the relationship, however, variable selection was based on multivariable analysis. |
| 4·6 Were complexities in the data (e.g., censoring, competing risks, sampling of controls) accounted for appropriately? | Yes | This model excludes 620 deaths that did not experience the study outcome. However, these deaths mostly occurred within the first 10 days of the study, thus carrying low risk to affect the predictive performance. |
| 4·7 Were relevant model performance measures evaluated appropriately? | Yes | Discrimination was assessed by C-statistics, and calibration was assessed by Hosmer-Lemeshow test. |
| 4·8 Were model overfitting and optimism in model performance accounted for? | Yes | Internal validation using the bootstrap method revealed the degree of over-optimism on c-statistics of the final prediction model was 0.0046, resulting in an equivalent c-statistic after bootstrap validation of 0.72. The final model changed little. |
| 4·9 Do predictors and their assigned weights in the final model correspond to the results from multivariable analysis? | Yes | Predictors and their assigned weights in the final model derived from the multivariable regression analysis. |
| **Overall risk of bias of Domain 4** | **Low risk of bias** |  |

Supplemental Figure S1. QR code for an online calculator based on the prediction model.


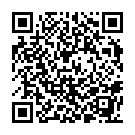


Note: The QR code can be scanned using mobile devices such as smart phones or iPad. The probability of risk will be shown automatically at completion of all predictors. Alternatively, readers can visit: http://redcap.scrds.net/surveys/ and enter the code: 7KMH8HN7N, to get this on-line calculator.
